# Washed microbiota transplantation is associated with *Helicobacter pylori*-negative conversion and circulating group 2 innate lymphoid cell profiles

**DOI:** 10.3389/fmed.2026.1869367

**Published:** 2026-08-20

**Authors:** Yi-Shu Wang, Yao-Fei Wei, Wei-Ran Chen, Zhi-Ning Ye, Hui-Min Zhou, Hao-Jie Zhong, Xing-Xiang He

**Affiliations:** 1 Department of Gastroenterology/Microbiota Medicine, Research Center for Engineering Techniques of Microbiota-Targeted Therapies of Guangdong Province, The First Affiliated Hospital of Guangdong Pharmaceutical University, Guangzhou, China; 2 Guangdong Provincial Key Laboratory for Research and Evaluation of Pharmaceutical Preparations, Guangdong Pharmaceutical University, Guangzhou, China; 3 The Department of Rheumatology, Shenzhen Traditional Chinese Medicine Hospital, The Fourth Clinical Medical College of Guangzhou University of Chinese Medicine, Shenzhen, China

**Keywords:** fecal microbiota transplantation, Helicobacter infections, *Helicobacter pylori*, innate lymphoid cells, peptic ulcer

## Abstract

**Background:**

Antibiotic resistance remains a major challenge in the management of *Helicobacter pylori* (*H. pylori*) infection. Microbiota-based interventions, including washed microbiota transplantation (WMT), have emerged as potential adjunctive strategies; however, their clinical outcomes in *H. pylori* infection and associated host immune alterations remain insufficiently characterized.

**Methods:**

This retrospective observational study included two independent cohorts from January 2020 to January 2025. The WMT outcome cohort comprised patients with baseline *H. pylori* positivity who completed WMT and had available post-treatment reassessment. Patients receiving prior or concomitant standard *H. pylori* eradication therapy were excluded. Post-WMT *H. pylori* status was assessed, and safety outcomes were recorded. The peripheral blood immune profiling cohort included individuals with confirmed *H. pylori* status and available peripheral blood samples. Circulating innate lymphoid cell (ILC) subsets, including ILC2s and integrin α4^+^ ILC2s, were analyzed by flow cytometry, and their associations with clinical phenotypes and post-WMT *H. pylori* status were explored.

**Results:**

The WMT cohort included 42 patients [mean age, 56.60 ± 14.65 years; 30 men (71.43%)]. During a median follow-up of 196.5 days (IQR, 44.5–595.0 days), 23 patients achieved post-WMT *H. pylori*-negative conversion, corresponding to an observed conversion proportion of 54.76% (23/42; exact 95% CI, 38.67–70.15%). Ninety-six WMT procedures were performed, with adverse events occurring in 5 procedures involving 5 patients. In the immune profiling cohort, patients with *H. pylori* infection exhibited higher circulating ILC2 and integrin α4^+^ ILC2 proportions and lower ILC1 proportions than healthy controls. Circulating ILC2-related parameters were associated with selected infection-related clinical features, including anti–*H. pylori* antibody levels and virulence-factor status. In available post-WMT immune samples, lower circulating ILC2 proportions were observed compared with available baseline samples, and patients with subsequent *H. pylori*-negative conversion showed lower circulating ILC2 proportions than those with persistent positivity.

**Conclusion:**

In this retrospective uncontrolled study, WMT exposure was associated with subsequent *H. pylori*-negative conversion and a low recorded adverse-event rate. Alterations in circulating ILC2-related profiles were associated with *H. pylori* infection status and post-WMT outcomes. These findings provide preliminary observational evidence supporting further investigation of WMT as an adjunctive microbiota-based strategy and of circulating ILC2-related parameters as potential immune biomarkers.

## 1 Introduction

*Helicobacter pylori* (*H. pylori*) infection is highly prevalent in China, with an estimated prevalence of approximately 40% (1). Since its discovery in 1982, *H. pylori* has been recognized as a major gastric pathogen. As a urease-producing, spiral-shaped bacterium, *H. pylori* can survive in the acidic gastric environment by producing ammonia to neutralize gastric acid, enabling persistent colonization of the gastric mucosa. Chronic *H. pylori* infection disrupts gastric physiological homeostasis and induces persistent inflammation, leading to gastric mucosal damage and the development of various gastric diseases (2). According to the Maastricht VI/Florence Consensus Report, *H. pylori* eradication strategies should be selected based on regional antibiotic resistance patterns and treatment efficacy (3). However, the increasing prevalence of antibiotic-resistant *H. pylori* strains worldwide has significantly reduced eradication rates and become a major obstacle to successful treatment (3, 4).

Evidence suggests that modulation of the gut microbiota, such as adjunctive probiotic supplementation, may enhance *H. pylori* eradication efficacy and reduce gastrointestinal symptoms or treatment-related adverse events when combined with standard therapy (5, 6). Accordingly, microbiota-based interventions, including fecal microbiota transplantation (FMT), have been investigated as potential adjunctive strategies for *H. pylori* eradication. Washed microbiota transplantation (WMT) represents a standardized modification of conventional FMT, in which automated filtration and repeated washing procedures are applied to remove fecal debris, residual metabolites, and non-target components while preserving a concentrated microbial community. Compared with manually prepared conventional FMT, this standardized processing approach may improve preparation consistency, traceability, and treatment tolerability (7, 8). However, preliminary clinical evidence remains limited. A pilot study demonstrated the feasibility of WMT in *H. pylori*–infected patients but failed to show a statistically significant eradication benefit, highlighting the need for validation in larger, controlled cohorts (9).

Group 2 innate lymphoid cells (ILC2s) exhibit bidirectional interactions with the gut microbiota and contribute to mucosal immune regulation (10–12). In the stomach, bacteria-induced ILC2s have been implicated in mucosal immune protection through IgA-associated responses (13), while ILC2-related type 2 immune pathways may participate in host responses to *H. pylori* infection (14). However, the characteristics and clinical relevance of ILC2 responses in *H. pylori* infection remain incompletely understood, particularly in cohorts integrating gastric function parameters, virulence factors, and microbiota-based interventions. Furthermore, whether WMT exposure is associated with alterations in circulating ILC2 profiles and whether these immune changes are related to subsequent *H. pylori* status remain unclear.

Therefore, this retrospective study aimed to investigate the association between WMT exposure and subsequent *H. pylori*–negative conversion, evaluate its safety profile, and characterize circulating ILC2-related immune responses in patients with *H. pylori* infection. We further explored the relationships between ILC2 profiles, clinical phenotypes, and post-WMT *H. pylori* status to provide preliminary insights into the potential immunological mechanisms underlying microbiota-based interventions.

## 2 Methods

### 2.1 Study design and participants

This retrospective observational study was conducted at the First Affiliated Hospital of Guangdong Pharmaceutical University. The study was designed to investigate the association between washed microbiota transplantation (WMT) exposure, subsequent *Helicobacter pylori* (*H. pylori*) status, and circulating innate lymphoid cell (ILC) profiles in patients with *H. pylori* infection.

Two independent cohorts were included in this study. The peripheral blood immune profiling cohort was established to characterize circulating ILC subsets in individuals with and without *H. pylori* infection. The WMT cohort was established to evaluate post-WMT *H. pylori* status, clinical outcomes, and safety profiles among patients with baseline *H. pylori* infection. The study protocol was approved by the Ethics Committee of the First Affiliated Hospital of Guangdong Pharmaceutical University (approval No. 2024–46), and written informed consent was obtained from all participants.

### 2.2 Definition and assessment of *Helicobacter pylori* infection status

Baseline *H. pylori* infection was defined based on at least one positive result from histopathological examination of gastric mucosal biopsy specimens, rapid urease testing, ^13^C-urea breath testing, or serum anti–*H. pylori* antibody testing. Positive serological results were considered evidence of baseline infection only in participants without a previous history of *H. pylori* eradication therapy.

For patients receiving WMT, post-WMT *H. pylori* status was assessed using the ^13^C-urea breath test. *H. pylori*–negative conversion was defined as a negative ^13^C-urea breath test result after WMT. Proton pump inhibitors were discontinued for at least 1 month before post-WMT *H. pylori* testing.

### 2.3 Peripheral blood ILC cohort and flow cytometric analysis

#### 2.3.1 Study population

Participants undergoing *H. pylori* testing between January 2020 and January 2025 were retrospectively screened for inclusion in the peripheral blood immune profiling cohort. Eligible participants were required to have definitive *H. pylori* status (positive or negative), available peripheral blood samples for immunophenotyping, and complete clinical information, including demographic characteristics and relevant laboratory parameters.

Participants were excluded if they had recent exposure to systemic corticosteroids or other immunosuppressive agents. The final immune profiling cohort included 128 participants, comprising 65 patients with *H. pylori* infection and 63 healthy controls.

#### 2.3.2 Flow cytometric analysis of peripheral blood ILC subsets

Peripheral blood mononuclear cells (PBMCs) were isolated using red blood cell lysis buffer (Solarbio, Beijing, China). Surface staining was performed using fluorochrome-conjugated monoclonal antibodies (BioLegend, San Diego, CA, USA). The lineage (Lin) cocktail included antibodies against CD3 (HIT3a), CD5 (UCHT2), CD11c (3.9), CD16 (B73.1), CD19 (HIB19), and TCRαβ (IP26). Additional antibodies included CD45 (H130), CD127 (A019D5), CD117 (A3C6E2), CD294 (BM16), and integrin α4 (CD49d; 9F10). Dead cells were excluded using 7-aminoactinomycin D (7-AAD). Data were acquired using a CytoFLEX flow cytometer (Beckman Coulter, Brea, CA, United States) and analyzed using CytoExpert software (version 2.3; Beckman Coulter). Within the lymphocyte gate, viable CD45^+^7-AAD^−^ cells were selected. ILCs were identified as Lin^−^CD127^+^ cells. ILC subsets were defined as follows: ILC1s (CD117^+^CD294^−^), ILC2s (CD117^−^CD294^+^), and ILC3s (CD117^+^CD294^+^).

### 2.4 WMT cohort and patient selection

To evaluate the association between WMT exposure and subsequent *H. pylori* status, patients receiving WMT at our institution during the study period were retrospectively screened. Patients were eligible if they met the following criteria: (1) baseline *H. pylori* positivity; (2) no previous history of *H. pylori* eradication therapy; (3) WMT as the primary study exposure; (4) completion of at least one WMT course; and (5) availability of post-WMT follow-up data allowing assessment of *H. pylori* status and/or gastric function parameters. Patients were excluded if they had received prior or concomitant standard *H. pylori* eradication therapy, including bismuth-containing quadruple therapy or other antibiotic-containing regimens. Patients who used systemic antibiotics or proton pump inhibitors within 1 month before WMT were also excluded. Importantly, WMT was performed primarily for clinical indications other than conventional *H. pylori* eradication therapy. For the present analysis, WMT was considered an exposure factor, and post-WMT *H. pylori* status was evaluated as an observational outcome.

### 2.5 Clinical data collection

Clinical data were retrospectively extracted from electronic medical records and laboratory information systems. For the peripheral blood immune profiling cohort, collected variables included demographic characteristics (age, sex, and body mass index [BMI]), gastric function-related parameters, including serum gastrin-17 (G-17), pepsinogen I (PGI), pepsinogen II (PGII), and the PGI/PGII ratio (PGR), anti–*H. pylori* antibody status, virulence factor status (CagA and VacA), and endoscopic findings, particularly peptic ulcer disease.

For the WMT cohort, additional clinical variables included smoking and alcohol history, WMT indications, delivery route, number of treatment courses, concomitant medications, WMT-related adverse events, and follow-up *H. pylori* status. WMT-related adverse events were identified retrospectively from medical records and included diarrhea and pharyngeal discomfort.

### 2.6 Washed microbiota transplantation procedure

WMT was performed according to the Nanjing consensus protocol. Healthy donors underwent comprehensive screening procedures, including questionnaires, physical examinations, blood analyses, and stool examinations. Eligible donor samples met Bristol Stool Form Scale types III–IV and were tracked using a traceable biospecimen coding system.

Briefly, 100 g of fresh donor stool was homogenized in 500 mL sterile normal saline (1:5, w/v), followed by automated microfiltration using an automated purification system (FMT Medical, Nanjing, China). The microbial suspension was centrifuged at 4 °C at 1100 × g for 3 min, washed three times with sterile saline, and resuspended in 100 mL saline to generate the final washed microbiota suspension.

WMT was administered at a dose of 120 mL/day for three consecutive days per treatment course. Delivery was performed through either a nasojejunal tube (upper gastrointestinal route) or an endoscopically placed enteral tube (lower gastrointestinal route). No antibiotic pretreatment was administered before WMT, and donor preparations were randomly allocated to recipients.

### 2.7 Statistical analysis

Statistical analyses were performed using SPSS version 25.0 (IBM, Armonk, NY, USA) and GraphPad Prism version 10.1.2 (GraphPad Software, San Diego, CA, USA). Continuous variables were presented as mean ± standard deviation (SD) or median [interquartile range (IQR)], according to data distribution. Categorical variables were presented as numbers and percentages. Comparisons between two independent groups were performed using the Student’s *t* test for normally distributed continuous variables or the Mann–Whitney U test for non-normally distributed variables. Paired continuous variables were compared using paired *t* test or Wilcoxon signed-rank test, as appropriate. Categorical variables were compared using Pearson’s chi-square test or Fisher’s exact test. The 95% confidence interval (CI) for the observed post-WMT *H. pylori*–negative conversion proportion was calculated using the exact binomial method. Correlations between continuous variables were assessed using Pearson or Spearman correlation analyses. For multiple comparisons, the Benjamini–Hochberg false discovery rate (FDR) correction was applied, and adjusted q values <0.05 were considered statistically significant. Exploratory binary logistic regression analysis was performed to identify potential factors associated with post-WMT *H. pylori*–negative conversion. Odds ratios (ORs) and corresponding 95% CIs were calculated. All statistical tests were two-sided.

## 3 Results

### 3.1 *Helicobacter pylori* status and safety outcomes after WMT

**Figure 1:**
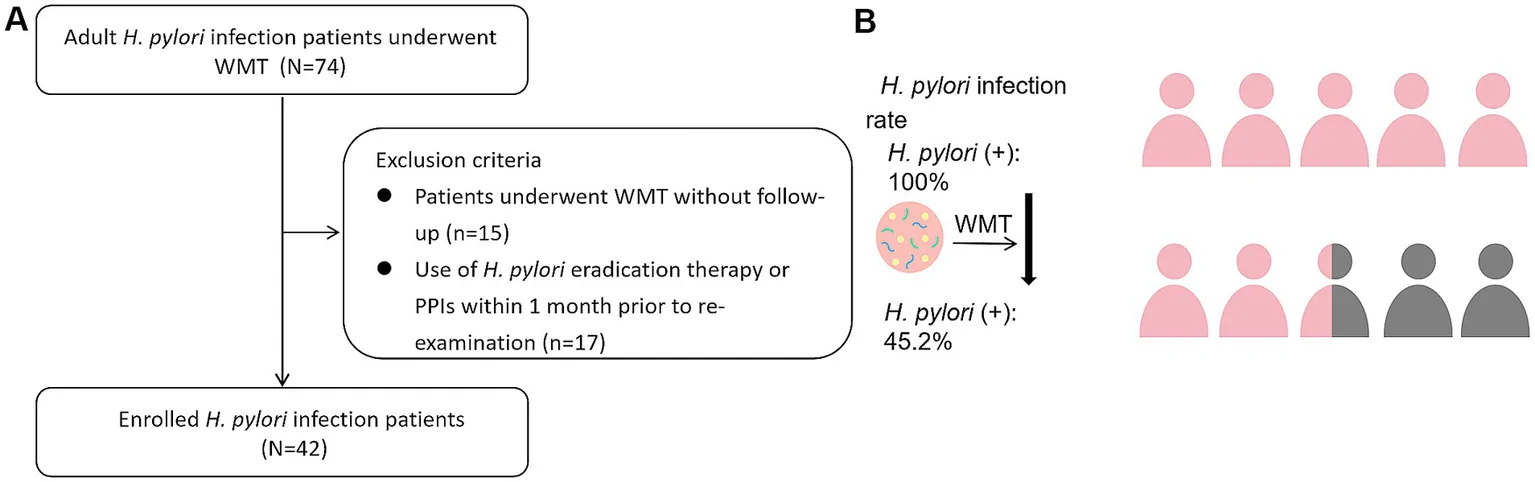
Study flowchart and changes in *Helicobacter pylori* status following washed microbiota transplantation (WMT). **(A)** Flowchart of patient enrollment and exclusion. **(B)** Changes in *H. pylori* status from baseline to the available post-WMT assessment.

A total of 42 patients with baseline *H. pylori* positivity who completed WMT and had available follow-up data were included according to the prespecified criteria. The study flow and changes in *H. pylori* status before and after WMT are shown in Figures 1A and 1B, respectively. Baseline demographic characteristics, diagnostic methods, gastric function indices, and WMT indications are summarized in Tables 1, 2. During a median follow-up of 196.5 days (IQR, 44.5–595.0 days), 23 patients achieved *H. pylori*–negative conversion confirmed by post-WMT ^13^C-urea breath testing, corresponding to a conversion proportion of 54.76% (23/42; exact 95% CI, 38.67–70.15%), whereas 19 patients remained *H. pylori* positive.

**Table 1:** Baseline demographic characteristics, diagnostic methods, and gastric function indicators of patients with *Helicobacter pylori* infection.

Characteristic	*H. pylori* infection (*n* = 42)
Age (years)	56.60 ± 14.65
Male sex, *n* (%)	30 (71.43)
BMI (kg/m^2^)	23.77 ± 4.30
Diagnosis of *H. pylori* infection, *n* (%)	
Histopathological evidence	10 (23.80)
Positive rapid urease test	15 (35.71)
Positive ^13^C-urea breath test	5 (11.90)
Positive anti-*H. pylori* serology (without prior eradication therapy)	12 (28.57)
Gastric function indicators (*n* = 36)	
G-17 (pmol/L)	2.82 (1.49, 7.35)
PGI (μg/L)	103.96 (77.92, 165.15)
PGII (μg/L)	11.42 (6.91, 17.07)
PGR	8.57 (6.44, 11.80)

Data are presented as mean ± standard deviation or median (interquartile range), as appropriate.

**Table 2:** Primary indications for washed microbiota transplantation in patients with *Helicobacter pylori* infection.

Primary cause of WMT	Number (*n*)	Percentage (%)
Functional bowel disease	27	64.29
Metabolic dysfunction-associated steatotic liver disease	4	9.52
Gastroesophageal reflux disease	3	7.14
Diabetes mellitus type 2	3	7.14
HBV-related cirrhosis	2	4.76
Gouty arthritis	1	2.38
Atopic dermatitis	1	2.38
Hyperlipidemia	1	2.38
Total	42	100

As functional bowel disease was the most common indication for WMT, we compared negative conversion rates between patients receiving WMT for functional bowel disease and those receiving WMT for other indications. Negative conversion occurred in 14 of 27 patients (51.9%) in the functional bowel disease group and 9 of 15 patients (60.0%) in the other-indication group, with no significant difference between groups (Fisher’s exact test, *p* = 0.750; Supplementary Table S1). Due to the limited number of patients with individual non-functional indications, further indication-specific analyses were not performed.

In an exploratory multivariable logistic regression model including sex, age ≥60 years, BMI ≥ 24 kg/m^2^, lower gastrointestinal delivery, smoking, and alcohol consumption, none of the evaluated variables was independently associated with post-WMT *H. pylori*–negative conversion. These findings should be interpreted cautiously given the limited sample size and wide confidence intervals (Supplementary Table S2).

In the gastric function analysis, ΔPGI showed a nominally significant difference between patients with and without post-WMT *H. pylori*–negative conversion. The median ΔPGI was −1.05 μg/L (IQR, −39.98 to 22.10 μg/L) in patients with negative conversion and 28.46 μg/L (IQR, −9.35 to 42.77 μg/L) in those with persistent *H. pylori* positivity (*p* = 0.030; Table 3 and Supplementary Table S3).

**Table 3:** Comparison of changes in gastric function indicators according to post-WMT *Helicobacter pylori* status.

Gastric function indicators	*H. pylori*-negative conversion (*n* = 17)	Persistent *H. pylori* positivity (*n* = 15)	*P* value
ΔG-17 (pmol/L)	−0.23 (−1.65, 2.77)	−0.16 (−1.39, 0.89)	0.823
ΔPGI (μg/L)	−1.05 (−39.98, 22.10)	28.46 (−9.35, 42.77)	0.030
ΔPGII (μg/L)	−1.24 (−6.29, 1.19)	0.74 (−2.50, 4.45)	0.246
ΔPGR	0.14 ± 6.60	−0.16 ± 5.34	0.889

Regarding safety, 96 WMT procedures were performed in 42 patients. Adverse events occurred in 5 procedures (procedure-level rate, 5.20%) involving 5 patients (patient-level rate, 11.90%). Recorded adverse events included pharyngeal discomfort after upper gastrointestinal tube placement in one patient and diarrhea in four patients. WMT was discontinued in the patient with pharyngeal discomfort and one patient with diarrhea; one patient with diarrhea improved after montmorillonite treatment and resumed WMT, while the remaining two patients recovered spontaneously.

### 3.2 Altered circulating ILC profiles associated with *Helicobacter pylori* infection

**Figure 2:**
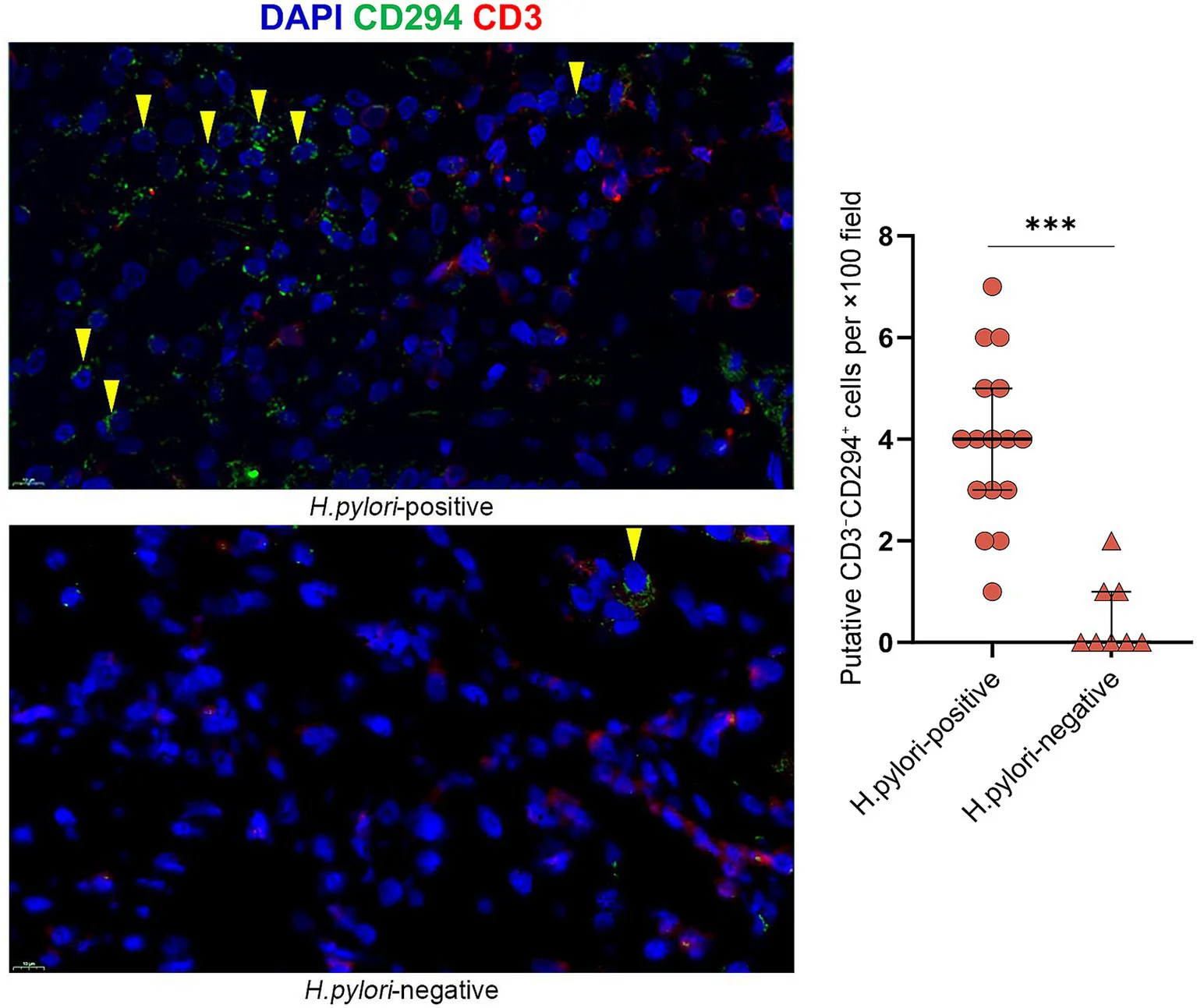
Exploratory immunofluorescence visualization and quantification of putative CD3^−^CD294^+^ cells in gastric mucosal specimens. Representative immunofluorescence images and quantitative analysis of CD3 and CD294 staining in gastric mucosal specimens from *H. pylori*-positive and *H. pylori*-negative participants are shown. Yellow arrows indicate putative CD3^−^CD294^+^ cells. The numbers of putative CD3^−^CD294^+^ cells were compared between *H. pylori*-positive (*n* = 16) and *H. pylori*-negative (*n* = 8) participants. Horizontal lines indicate the median and interquartile range. Comparisons were performed using the two-sided Mann–Whitney U test. ***p < 0.001. Scale bars = 10 μm.

Exploratory immunofluorescence staining was performed to characterize CD3^−^CD294^+^ cells in gastric mucosal specimens (Figure 2). Quantitative analysis of 16 *H. pylori*-positive and 8 *H. pylori*-negative participants revealed a higher abundance of putative CD3^−^CD294^+^ cells in *H. pylori*-positive participants [4.0 (3.0, 5.0) vs. 0.0 (0.0, 1.0) cells/field; Mann–Whitney U = 3, *p* < 0.001] (Figure 2).

**Figure 3:**
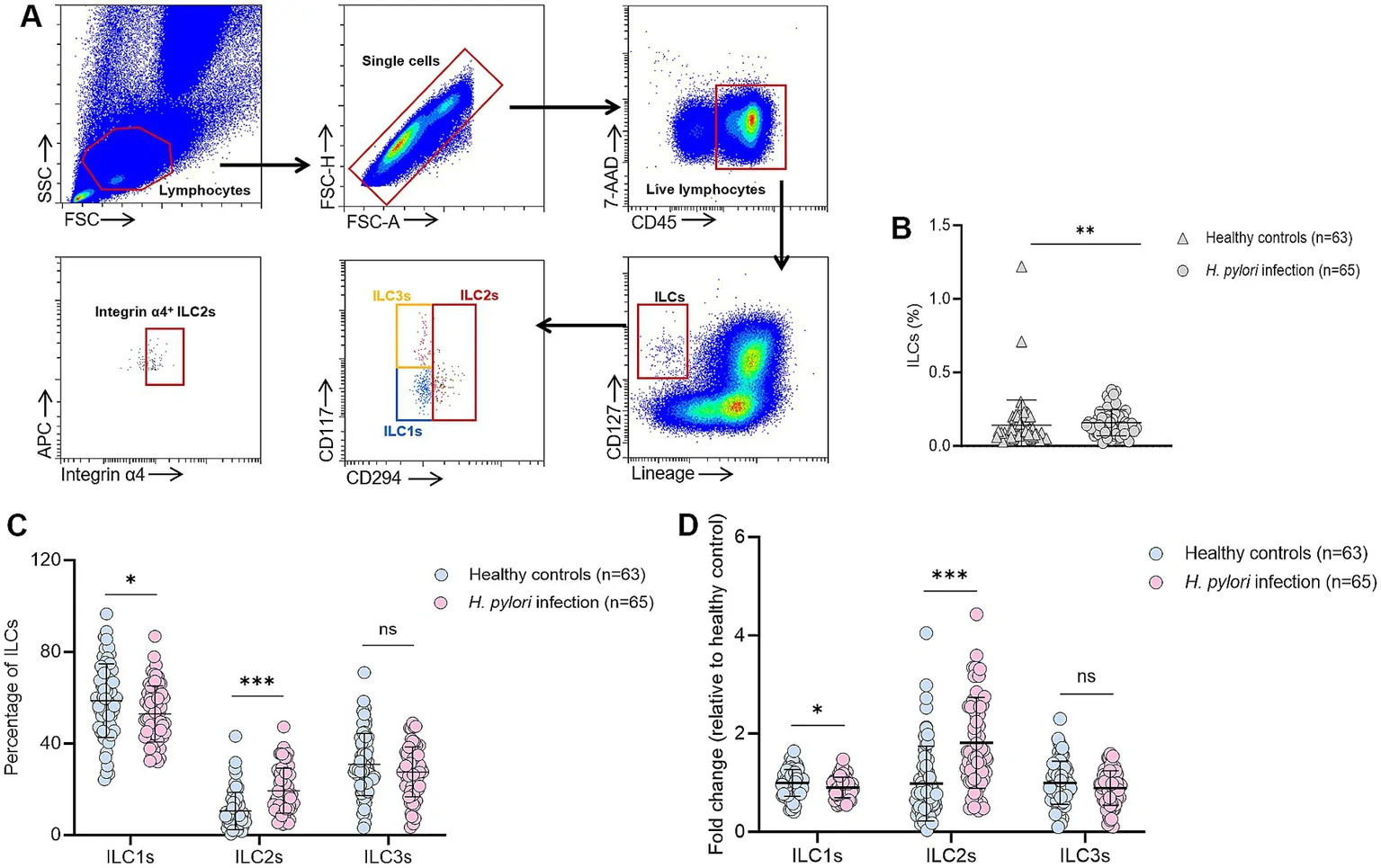
Alterations in circulating innate lymphoid cells (ILCs) in patients with *H. pylori* infection. **(A)** Gating strategy for the identification of circulating ILCs and ILC subsets by flow cytometry. **(B)** Comparison of the proportion of total circulating ILCs between healthy controls and patients with *H. pylori* infection. **: *p* < 0.01. **(C)** Comparison of ILC subsets (ILC1s, ILC2s, and ILC3s) between healthy controls and patients with *H. pylori* infection **(D)** Fold change of ILC subsets in patients with *H. pylori* infection relative to healthy controls. ILCs, innate lymphoid cells; ILC1s, type 1 innate lymphoid cells; ILC2s, type 2 innate lymphoid cells; ILC3s, type 3 innate lymphoid cells. *: *p* < 0.05; ***: *p* < 0.001; ns, not significant.

In peripheral blood, patients with *H. pylori* infection exhibited increased proportions of total ILCs compared with healthy controls (0.14 [0.09, 0.20] vs. 0.10 [0.06, 0.18]; *p* = 0.010) (Figure 3B). Among ILC subsets, ILC1s were decreased (52.96 ± 12.28 vs. 58.72 ± 16.11;*p* = 0.024), whereas ILC2s were increased (16.54 [11.76, 27.15] vs. 8.41 [5.04, 13.51]; *p* < 0.001). No significant difference was observed in ILC3 proportions (27.57 ± 10.84 vs. 30.81 ± 13.54; *p* = 0.136) (Figure 3C). Fold-change analysis identified ILC2s as the most prominently altered ILC subset (Figure 3D and Supplementary Table S4).

### 3.3 Circulating ILC2s reflect *Helicobacter pylori*-related clinical features

**Figure 4:**
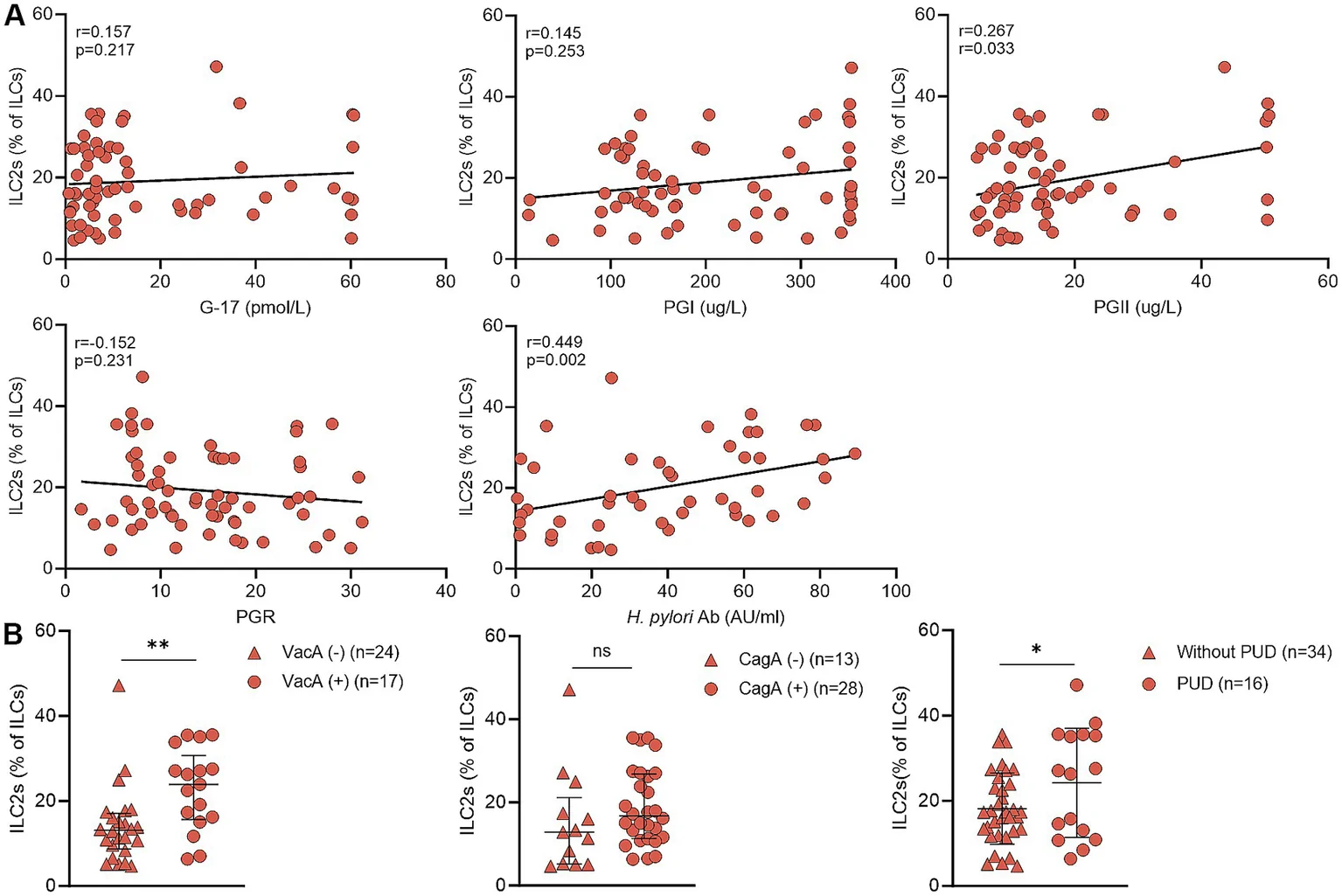
Association of circulating ILC2 proportions with gastric function indicators and *H. pylori*–related factors. **(A)** Correlation analyses between circulating ILC2 proportions and gastric function indicators. **(B)** Comparison of circulating ILC2 proportions according to VacA status, CagA status, and the presence of peptic ulcer disease. Data are presented as mean ± standard deviation or median (interquartile range), as appropriate. Significance symbols indicate nominal *p* values before FDR correction: *: *p* < 0.05; **: *p* < 0.01; ns, not significant. Corresponding FDR-adjusted *q* values are reported in the supplementary tables.

Within the *H. pylori*-infected cohort, circulating ILC2 proportions were positively correlated with anti–*H. pylori* antibody levels (*r* = 0.449, *p* = 0.002). A weak positive correlation was also observed between ILC2 proportions and PGII (*r* = 0.267, *p* = 0.033), whereas no significant correlations were detected with G-17, PGI, or PGR (Figure 4A and Supplementary Table S5).

Patients with VacA-positive infection exhibited higher circulating ILC2 proportions than those with VacA-negative infection (*p* = 0.003). No significant differences in circulating ILC2 proportions were observed according to CagA status. Patients with peptic ulcer disease showed a higher circulating ILC2 proportion than those without peptic ulcer disease (*p* = 0.049); however, this difference was modest (Figure 4B and Supplementary Table S6).

### 3.4 Integrin α4^+^ ILC2s are associated with gastric immune-related phenotypes

Integrin α4 (CD49d) is an adhesion and trafficking molecule expressed on immune cells (15). α4-containing integrin’s mediate leukocyte recruitment to gastrointestinal mucosal tissues through interactions with vascular and mucosal addressins, including VCAM-1 and MAdCAM-1. However, whether circulating integrin α4^+^ ILC2s undergo altered trafficking or tissue redistribution during *H. pylori* infection remains unclear.

**Figure 5:**
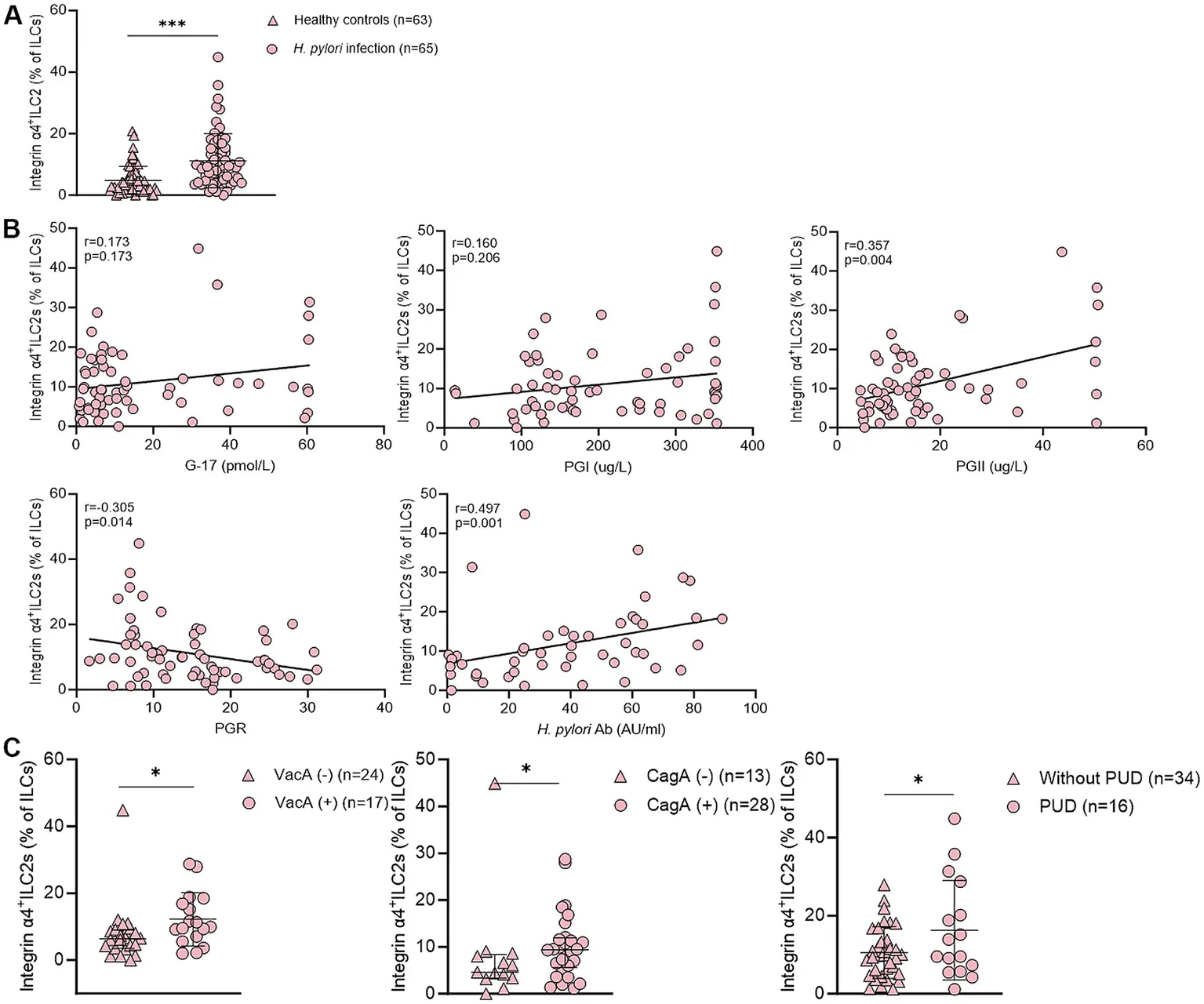
Circulating integrin α4^+^ ILC2 proportions in patients with *H. pylori* infection and healthy controls. **(A)** Comparison of circulating integrin α4^+^ ILC2 proportions between healthy controls and patients with *H. pylori* infection. **(B)** Correlation analyses between circulating integrin α4^+^ ILC2 proportions and gastric function indicators. **(C)** Comparison of circulating integrin α4^+^ ILC2 proportions according to *H. pylori* virulence factors and the presence or absence of peptic ulcer disease. Significance symbols indicate nominal p values before FDR correction: *: *p* < 0.05; **: *p* < 0.01; ns, not significant. Corresponding FDR-adjusted *q* values are reported in the supplementary tables.

Patients with *H. pylori* infection exhibited a higher proportion of circulating integrin α4^+^ ILC2s among total ILCs compared with healthy controls (9.33 [4.91, 15.26] [*n* = 65] vs. 3.27 [1.78, 6.83] [*n* = 63]; *p* < 0.001) (Figure 5A and Supplementary Table S4).

Within the *H. pylori*-infected cohort, circulating integrin α4^+^ ILC2 proportions were positively correlated with PGII (*r* = 0.357, *p* = 0.004) and anti–*H. pylori* antibody levels (*r* = 0.497, *p* = 0.001), but negatively correlated with PGR (*r* = −0.305, *p* = 0.014). No significant correlations were observed with G-17 (*r* = 0.173, *p* = 0.173) or PGI (*r* = 0.160, *p* = 0.206) (Figure 5B and Supplementary Table S7).

The circulating integrin α4^+^ ILC2 proportion was higher in patients with VacA-positive infection than in those with VacA-negative infection (9.91 [6.26, 17.66] [*n* = 17] vs. 6.31 [3.44, 8.96] [*n* = 24]; *p* = 0.012). Similarly, patients with CagA-positive infection showed a higher proportion of circulating integrin α4^+^ ILC2s than those with CagA-negative infection (9.42 [5.60, 11.93] [*n* = 28] vs. 4.66 [3.30, 8.34] [*n* = 13]; *p* = 0.036). Among the 50 patients undergoing endoscopy, those with peptic ulcer disease (*n* = 16) exhibited a higher circulating integrin α4^+^ ILC2 proportion than those without peptic ulcer disease (*n* = 34) (16.30 ± 12.75 vs. 10.57 ± 6.63; *p* = 0.041) (Figure 5C and Supplementary Table S6).

### 3.5 Post-WMT changes in circulating ILC2-related profiles

Emerging evidence suggests that the gut microbiota can modulate ILC2 biology; however, circulating ILC2 profiles after microbiota-based interventions in patients with *H. pylori* infection remain poorly characterized. In this exploratory analysis, immune profiling data were available for 23 patients at baseline and 16 patients after WMT. This cohort was distinct from the 42-patient WMT outcome cohort, with only three overlapping patients. Because the samples were not fully paired and the subgroup size was limited, these findings were interpreted as descriptive between-group associations rather than definitive longitudinal changes.

**Figure 6:**
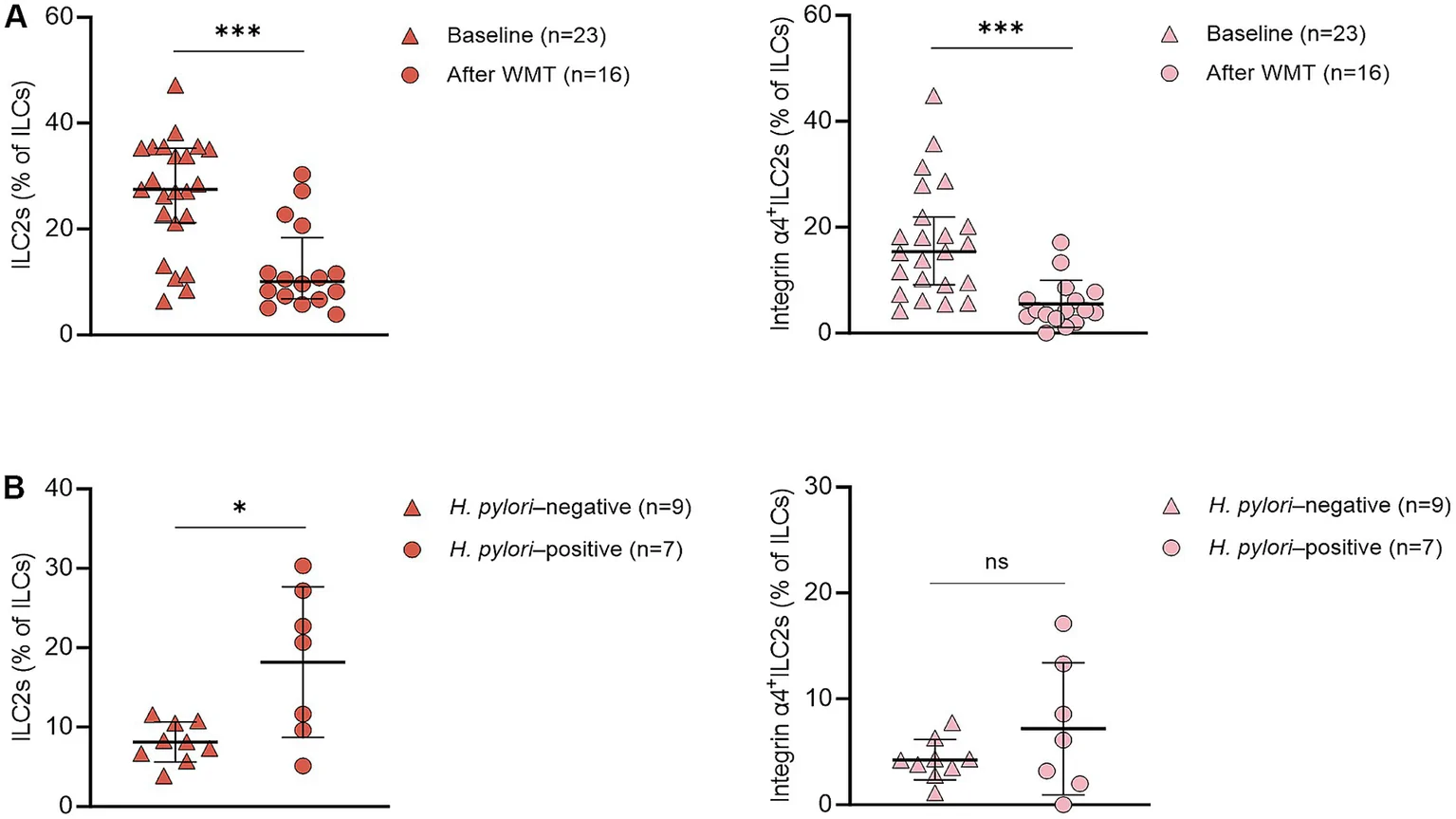
Circulating ILC2 and integrin α4^+^ ILC2 profiles in available baseline and post-WMT samples. **(A)** Proportions of circulating ILC2s and integrin α4^+^ ILC2s in available baseline and post-WMT samples. **(B)** Proportions of circulating ILC2s and integrin α4^+^ ILC2s according to post-WMT *H. pylori* status. Significance symbols indicate nominal *p* values before FDR correction: *P: < 0.05; **P: < 0.01; ns, not significant. Corresponding FDR-adjusted q values are reported in the Supplementary Tables.

The circulating ILC2 proportion was lower in available post-WMT samples than in available baseline samples (10.08 [6.83, 18.40] vs. 27.48 [21.16, 35.29]; *p* < 0.001). Similarly, the circulating integrin α4^+^ ILC2 proportion was reduced in post-WMT samples compared with baseline samples (4.27 [2.89, 7.40] vs. 15.37 [9.12, 21.92]; *p* < 0.001) (Figure 6A and Supplementary Table S8).

Among patients with available post-WMT immune data, those with *H. pylori*–negative conversion showed a lower circulating ILC2 proportion than those with persistent positivity (8.10 ± 2.53 [*n* = 9] vs. 18.18 ± 9.50 [*n* = 7]; *p* = 0.031). In contrast, the circulating integrin α4^+^ ILC2 proportion did not differ significantly between the two groups (4.24 ± 1.91 [*n* = 9] vs. 7.18 ± 6.24 [*n* = 7]; *p* = 0.269) (Figure 6B and Supplementary Table S8). These exploratory findings suggest an association between post-WMT *H. pylori* status and circulating ILC2 profiles but do not establish that WMT directly reduced circulating ILC2 levels.

## 4 Discussion

**Figure 7:**
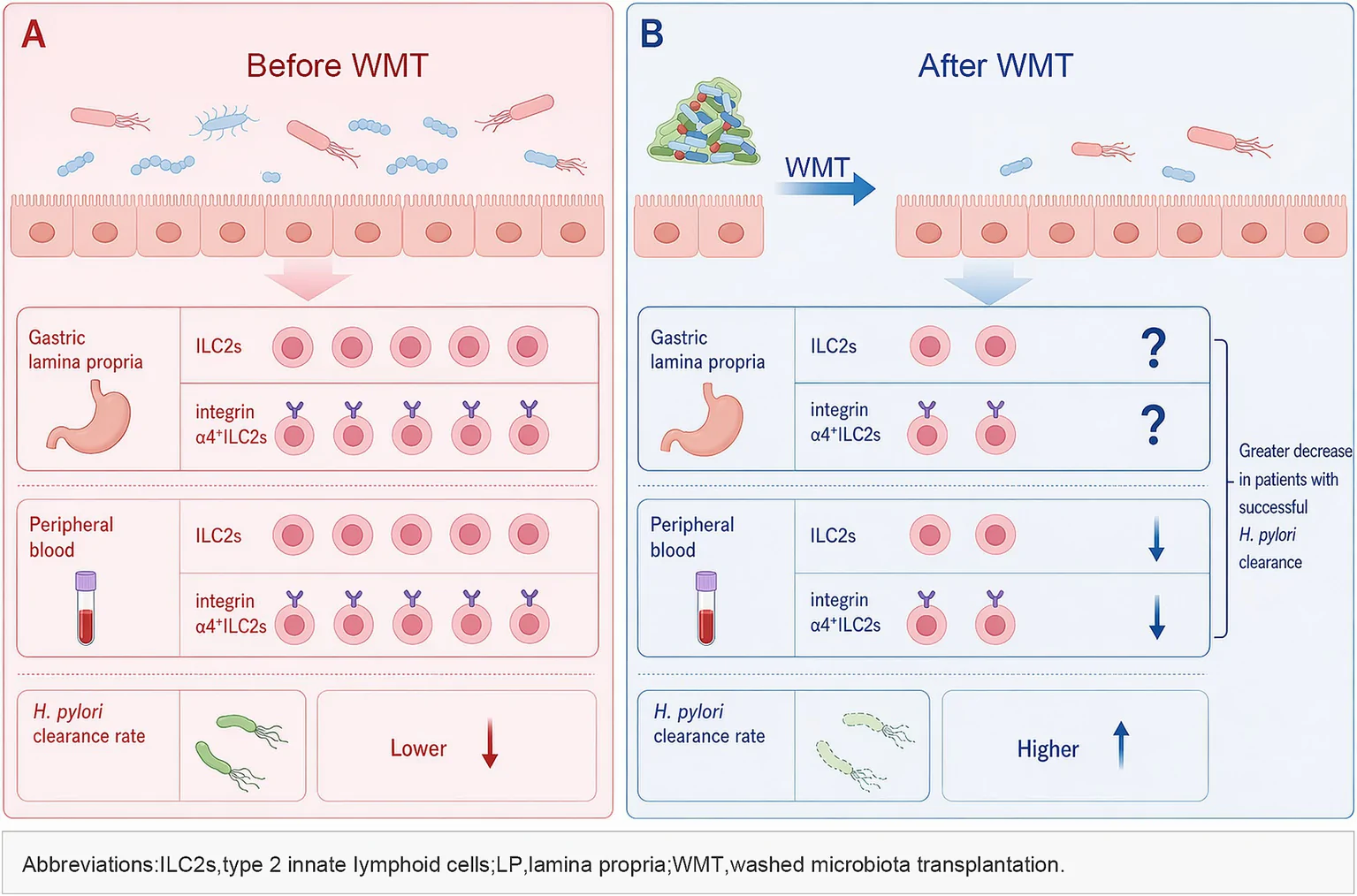
Conceptual framework illustrating potential microbiota–immune interactions associated with WMT exposure and *Helicobacter pylori* status. This study observed that WMT exposure was associated with subsequent *H. pylori*–negative conversion in a subset of patients and identified altered circulating ILC2-related profiles associated with *H. pylori* infection and post-WMT status. **(A)** Association between WMT exposure and subsequent *H. pylori*-negative conversion. **(B)** Association between *H. pylori* status, circulating ILC2-related profiles, and potential microbiota–immune regulation.

In this retrospective uncontrolled study, we observed *H. pylori*-negative conversion after WMT exposure in a subset of infected patients and characterized circulating ILC-related immune profiles in relation to *H. pylori* infection and post-WMT status. Patients with *H. pylori* infection exhibited increased circulating ILC2 and integrin α4^+^ ILC2 proportions compared with healthy controls, and these immune profiles were associated with selected clinical phenotypes. In available post-WMT immune samples, lower circulating ILC2-related proportions were observed, particularly among patients with subsequent *H. pylori*-negative conversion (Figure 7). These findings provide preliminary evidence linking microbiota-based intervention, *H. pylori* status, and host immune responses; however, they do not establish the therapeutic efficacy of WMT or a causal microbiota–immune mechanism (9, 13, 16).

The clinical significance of WMT in *H. pylori* infection remains exploratory. Our previous pilot study suggested that WMT might facilitate *H. pylori*-negative conversion, although statistical significance was not achieved, likely because of the limited sample size (9). In the present expanded cohort, more than half of the patients showed subsequent *H. pylori*-negative conversion after WMT, providing additional real-world observational evidence. However, these findings should not be interpreted as demonstrating that WMT can replace standard antimicrobial eradication therapy. Current evidence supporting microbiota-based approaches for *H. pylori* management remains insufficient, and standard antibiotic-based eradication remains the recommended therapeutic strategy (3, 17). Previous studies of probiotics have mainly supported their use as adjuncts to conventional eradication regimens by improving eradication rates or reducing treatment-related adverse events (18–25), whereas probiotic monotherapy has shown limited efficacy, with only transient suppression of *H. pylori* colonization and insufficient eradication rates to replace antibiotic-based therapy (26). Compared with probiotics or synbiotics, WMT introduces a more complex microbial community and may theoretically provide broader ecological restoration (7, 8). Nevertheless, direct comparative evidence demonstrating superiority of WMT over other microbiota-based interventions is currently unavailable. Therefore, WMT should be considered an investigational adjunctive strategy, and prospective controlled studies are required to determine its clinical value in *H. pylori* management.

The mechanisms underlying potential microbiota-mediated effects in *H. pylori* infection remain incompletely understood (27). Beyond direct antimicrobial activity, microbiota-based interventions may influence disease outcomes through restoration of microbial ecological balance and modulation of host mucosal immunity (28, 29). In this context, our study provides additional immunological observations by identifying alterations in circulating ILC2-related profiles associated with *H. pylori* infection and post-WMT status. These findings support the possibility that host immune responses may represent an important component of microbiota–host interactions during *H. pylori* infection.

ILC2s are increasingly recognized as regulators of mucosal immunity and exhibit bidirectional interactions with the microbiota (30). Previous studies demonstrated that bacteria-induced gastric ILC2s can contribute to mucosal defense through IL-5-dependent IgA responses, suggesting a protective role of ILC2s in maintaining gastric immune homeostasis (13). Conversely, *H. pylori*-associated inflammatory environments may activate ILC2-related type 2 immune responses through mechanisms involving macrophage polarization and epithelial-derived cytokine signaling (Supplementary Figure S1) (14, 16). Consistent with previous observations, we found that patients with *H. pylori* infection exhibited higher circulating ILC2 proportions than healthy controls, suggesting that ILC2-associated immune alterations are not restricted to the gastric mucosal compartment but may also be reflected systemically (16). Exploratory gastric mucosal staining further showed increased numbers of putative CD3^−^CD294^+^ cells in *H. pylori*-positive participants, providing additional support for potential local ILC2 enrichment. However, these findings require cautious interpretation because the staining strategy did not include comprehensive lineage markers and was based on limited tissue sampling.

The clinical associations observed in our cohort further suggest that circulating ILC2s may reflect aspects of *H. pylori*-related immune activation. The positive correlation between ILC2 proportions and anti–*H. pylori* antibody levels, together with higher ILC2 proportions in patients with VacA-positive infection, suggests that ILC2 responses may be related to bacterial exposure or inflammatory characteristics. However, these associations should not be interpreted as evidence that ILC2s directly determine disease severity, because the present study was observational and did not assess functional immune responses. Future studies integrating cytokine profiling, tissue-level immune characterization, and microbiome analysis are needed to clarify whether ILC2s represent drivers, markers, or consequences of *H. pylori*-associated inflammation.

Integrin α4^+^ ILC2s represent another potentially relevant immune phenotype identified in this study. Integrin α4 is an adhesion molecule involved in immune-cell trafficking, particularly through α4β7-mediated interactions with mucosal addressins such as MAdCAM-1 (15, 31). Previous studies have demonstrated that lymphocytes activated by gastric mucosal antigens can express mucosal homing-associated integrins and that *H. pylori* infection is accompanied by increased expression of MAdCAM-1 and lymphocyte recruitment within gastric tissues (15, 31, 32). In our cohort, circulating integrin α4^+^ ILC2 proportions were increased in *H. pylori*-infected individuals and were associated with several infection-related features, including antibody levels, virulence-factor status, and peptic ulcer disease. These findings raise the possibility that integrin α4^+^ ILC2s represent a phenotypically distinct circulating ILC2 population associated with mucosal immune activation. Nevertheless, integrin α4 expression alone cannot establish tissue homing capacity, and the present peripheral blood analyses cannot demonstrate direct migration or functional participation of these cells within gastric mucosa.

The relationship between microbiota modulation and ILC2 responses remains an emerging area of investigation. Experimental studies have shown that microbial composition and microbial metabolites can influence ILC2 abundance and function through epithelial sensing pathways and metabolite-mediated signaling networks. For example, tuft cell-derived IL-25 regulates intestinal ILC2 homeostasis, while microbial metabolites such as succinate and short-chain fatty acids can modulate ILC2-related responses in a context-dependent manner (Supplementary Figure S1) (11, 12, 27–29). In the present study, available post-WMT samples showed lower circulating ILC2 and integrin α4^+^ ILC2 proportions compared with available baseline samples, and lower ILC2 proportions were observed among patients with post-WMT *H. pylori*-negative conversion. However, these findings should be interpreted as exploratory associations rather than evidence that WMT directly reduced ILC2 responses. Importantly, the immune profiling cohort was distinct from the 42-patient WMT outcome cohort, with only three overlapping patients, and immune samples were not fully paired. Moreover, microbiome composition, microbial metabolites, cytokines, and tissue immune responses were not simultaneously assessed. Therefore, whether WMT modifies *H. pylori* status through microbiota-driven regulation of ILC2 responses remains an important hypothesis requiring prospective mechanistic validation.

This study has several strengths. Unlike previous microbiota-based studies in *H. pylori* infection that mainly focused on clinical outcomes, we integrated microbiota intervention assessment with circulating immune-cell profiling and clinical phenotyping. This approach provides preliminary insights into potential host immune responses associated with microbiota-based strategies. In addition, the expanded WMT cohort allowed further evaluation of post-WMT *H. pylori* status compared with previous pilot observations.

Several limitations should be acknowledged. First, the retrospective single-center design and lack of a control group preclude causal inference regarding the clinical effects of WMT. Second, the WMT cohort was clinically heterogeneous, follow-up duration varied among patients, and selection bias may have been introduced because inclusion required available post-WMT *H. pylori* reassessment, while patients with incomplete follow-up or concomitant eradication therapy were excluded, which may limit the generalizability of the findings. Third, immune analyses were exploratory, with incomplete longitudinal sampling and limited tissue-level characterization. Finally, microbiome composition, microbial metabolites, and functional immune mechanisms were not evaluated, preventing direct assessment of microbiota-mediated pathways underlying the observed associations. Future prospective multicenter studies integrating microbiome, metabolomic, and immune profiling are warranted.

In conclusion, this retrospective study suggests that WMT exposure was associated with subsequent *H. pylori*-negative conversion and a relatively low recorded adverse-event rate, although causal efficacy remains to be established. *H. pylori* infection was associated with increased circulating ILC2 and integrin α4^+^ ILC2 proportions, while post-WMT *H. pylori* status was associated with differences in circulating ILC2 profiles. These findings suggest that circulating ILC2-related parameters may represent candidate biomarkers for further investigation and provide a potential immunological framework for understanding microbiota-based interventions in *H. pylori* infection. Prospective controlled studies integrating microbiome, metabolomic, and tissue-level immune analyses are warranted to clarify the clinical role of WMT and the mechanisms linking microbial ecology with mucosal immunity.

## Data Availability

The raw data supporting the conclusions of this article will be made available by the authors, without undue reservation.
