# Supplementary material for: Washed microbiota transplantation is associated with Helicobacter pylori-negative conversion and circulating group 2 innate lymphoid cell profiles
Source: Front Med (Lausanne). 2026 Aug 20;13:1869367. doi: 10.3389/fmed.2026.1869367 (PMC13540426; doi:10.3389/fmed.2026.1869367)
Supplement: Supplementary file 1 [file Supplementary_file_1.docx]

**Supplementary Materials**

Supplementary Table S1. Association between the primary clinical indication for WMT and post-WMT *H. pylori*-negative conversion.
Supplementary Table S2. Exploratory multivariable logistic regression analysis of factors associated with post-WMT *H. pylori*-negative conversion.
Supplementary Table S3. Exploratory comparison of changes in gastric function indices according to post-WMT *H. pylori* status.
Supplementary Table S4. Comparison of circulating innate lymphoid cell profiles between healthy controls and patients with Helicobacter pylori infection.
Supplementary Table S5. Exploratory correlation analyses between circulating ILC2 proportions and gastric function indicators or anti–*H. pylori* antibody levels.
Supplementary Table S6. Exploratory subgroup comparisons of circulating ILC2-related parameters according to Helicobacter pylori virulence-factor status and peptic ulcer disease.
Supplementary Table S7. Exploratory correlation analyses between circulating integrin α4⁺ ILC2 proportions and gastric function indicators or anti–Helicobacter pylori antibody levels.
Supplementary Table S8. Exploratory comparisons of circulating ILC2-related profiles between available baseline and post-WMT samples and according to post-WMT *H. pylori* status.

Supplementary Figure S1. Hypothetical model illustrating potential microbiota–ILC2 interactions in Helicobacter pylori infection and washed microbiota transplantation (WMT)-related immune responses.

**Supplementary Table S1. Association between the primary clinical indication for WMT and post-WMT Helicobacter pylori-negative conversion.**

| WMT indication | Persistent *H. pylori* positivity, n (%) | *H. pylori-*negative conversion, n (%) | Total | P value |
| --- | --- | --- | --- | --- |
| Functional bowel disease | 13 (48.1%) | 14 (51.9%) | 27 | 0.750 |
| Non-functional bowel disease | 6 (40.0%) | 9 (60.0%) | 15 |  |
| Total | 19 (45.2%) | 23 (54.8%) | 42 |  |

The association between the primary indication for WMT and post-WMT *H. pylori*-negative conversion was assessed using Fisher’s exact test. WMT, washed microbiota transplantation.

**Supplementary Table S2. Exploratory multivariable logistic regression analysis of factors associated with post-WMT Helicobacter pylori-negative conversion.**

| Variable | OR (95%CI) | P value |
| --- | --- | --- |
| Male sex | 0.93 (0.20-4.30) | 0.931 |
| Age≥60 years (vs <60 years) | 0.75 (0.19–3.01) | 0.684 |
| BMI≥24kg/m² (vs＜24kg/m²) | 0.55 (0.15–2.03) | 0.368 |
| Smoking history | 2.50 (0.31–19.90) | 0.388 |
| Alcohol consumption | 1.59 (0.09–29.66) | 0.757 |
| Lower gastrointestinal delivery route  (vs upper/non-lower route) | 1.13 (0.27–4.66) | 0.867 |

Post-WMT *H. pylori*-negative conversion was entered as the dependent variable and coded as 1; persistent positivity was coded as 0. All covariates were entered simultaneously using the Enter method. Adjusted odds ratios and 95% confidence intervals were obtained from the multivariable logistic regression model. The analysis was considered exploratory because of the limited sample size and number of outcome events. OR, odds ratio; CI, confidence interval; BMI, body mass index; WMT, washed microbiota transplantation.

**Supplementary Table S3. Exploratory comparison of changes in gastric function indices according to post-WMT Helicobacter pylori status.**

| Compared indicator | *H. pylori*-negative conversion (n=17) | *Persistent H. pylori positivity*  (n=15) | Nominal  P value | FDR-adjusted q value |
| --- | --- | --- | --- | --- |
| ΔG-17 (pmol/L) | -0.23 (-1.65, 2.77) | -0.16 (-1.39, 0.89) | 0.823 | 0.889 |
| ΔPGI (µg/L) | -1.05 (-39.98, 22.10) | 28.46 (-9.35, 42.77) | 0.030 | 0.120 |
| ΔPGII (µg/L) | -1.24 (-6.29, 1.19) | 0.74 (-2.50, 4.45) | 0.246 | 0.491 |
| ΔPGR | 0.14 ± 6.60 | -0.16 ± 5.34 | 0.889 | 0.889 |

Values are presented as mean ± standard deviation or median (interquartile range), as appropriate. Δ was calculated as the post-WMT value minus the baseline value. Nominal P values were obtained using the independent-samples Student’s t test or Mann–Whitney U test according to data distribution. FDR-adjusted q values were calculated using the Benjamini–Hochberg procedure within this prespecified gastric-function comparison family. G-17, gastrin-17; PGI, pepsinogen I; PGII, pepsinogen II; PGR, PGI/PGII ratio; FDR, false discovery rate.

**Supplementary Table S4. Comparison of circulating innate lymphoid cell profiles between healthy controls and patients with Helicobacter pylori infection.**

| Compared indicator | Healthy controls  (n = 63) | *H. pylori* infection  (n = 65) | Nominal P value | FDR-adjusted  q value |
| --- | --- | --- | --- | --- |
| Total ILCs | 0.10 (0.06, 0.18) | 0.14 (0.09, 0.20) | 0.010 | 0.016 |
| ILC1s/ILCs | 58.72±16.11 | 52.96±12.28 | 0.024 | 0.030 |
| ILC2s/ILCs | 8.41 (5.04, 13.51) | 16.54 (11.76, 27.15) | <0.001 | <0.001 |
| ILC3s/ILCs | 30.81±13.54 | 27.57±10.84 | 0.136 | 0.136 |
| Integrin α4^⁺^ ILC2s/ILCs | 3.27 (1.78, 6.83） | 9.33 (4.91, 15.26) | <0.001 | <0.001 |

Values are presented as mean ± standard deviation or median (interquartile range), as appropriate. Nominal P values were obtained using the independent-samples Student’s t test or Mann–Whitney U test according to data distribution. FDR-adjusted q values were calculated using the Benjamini–Hochberg procedure within this prespecified between-group comparison family. ILC, innate lymphoid cell; FDR, false discovery rate.

**Supplementary Table S5. Exploratory correlation analyses between circulating ILC2 proportions and gastric function indicators or anti–Helicobacter pylori antibody levels.**

| ILC2-related parameter | Clinical variable | Correlation coefficient | Nominal  P value | FDR-adjusted q value |
| --- | --- | --- | --- | --- |
| ILC2s/ILCs | G-17 | 0.157 | 0.217 | 0.253 |
| ILC2s/ILCs | PGI | 0.145 | 0.253 | 0.253 |
| ILC2s/ILCs | PGII | 0.267 | 0.033 | 0.083 |
| ILC2s/ILCs | PGR | -0.152 | 0.231 | 0.253 |
| ILC2s/ILCs | Anti-*H. pylori* antibody | 0.449 | 0.002 | 0.010 |

Correlations were assessed using Pearson correlation analysis or Spearman rank correlation analysis, as appropriate. FDR-adjusted q values were calculated using the Benjamini–Hochberg procedure within this prespecified ILC2 correlation-analysis family. The correlation coefficient is reported as Pearson’s r or Spearman’s ρ, according to the method used. G-17, gastrin-17; PGI, pepsinogen I; PGII, pepsinogen II; PGR, PGI/PGII ratio; FDR, false discovery rate.

**Supplementary Table S6. Exploratory subgroup comparisons of circulating ILC2-related parameters according to Helicobacter pylori virulence-factor status and peptic ulcer disease.**

| Compared indicator | Group A | Group B | Nominal  P value | FDR-adjusted  q value |
| --- | --- | --- | --- | --- |
| ILC2s/ILCs | VacA-positive:  23.90 (15.64, 30.69)  n=17 | VacA-negative:  13.09 (8.71, 17.07)  n=24 | 0.003 | 0.015 |
| Integrin α4^⁺^ ILC2s/ILCs | VacA-positive:  9.91 (6.26, 17.66)  n=17 | VacA-negative:  6.31 (3.44, 8.96)  n=24 | 0.012 | 0.037 |
| ILC2s/ILCs | CagA-positive:  16.76 (11.40, 26.88) n=28 | CagA-negative:  12.85 (5.22, 21.21) n=13 | 0.121 | 0.121 |
| Integrin α4^⁺^ ILC2s/ILCs | CagA-positive:  9.42 (5.60, 11.93)  n=28 | CagA-negative:  4.66 (3.30, 8.34)  n=13 | 0.036 | 0.059 |
| ILC2s/ILCs | Peptic ulcer disease: 24.22±12.82  n=16 | No peptic ulcer disease:  18.13±8.360  n=34 | 0.049 | 0.059 |
| Integrin α4^⁺^ ILC2s/ILCs | Peptic ulcer disease: 16.30 ± 12.75  n=16 | No peptic ulcer disease: 10.57 ± 6.631  n=34 | 0.041 | 0.059 |

Values are presented as mean ± standard deviation or median (interquartile range), as appropriate. Nominal P values were obtained using the independent-samples Student’s t test or Mann–Whitney U test according to data distribution. FDR-adjusted q values were calculated using the Benjamini–Hochberg procedure within this prespecified clinical-subgroup comparison family. VacA, vacuolating cytotoxin A; CagA, cytotoxin-associated gene A; ILC2, group 2 innate lymphoid cell; FDR, false discovery rate.

**Supplementary Table S7. Exploratory correlation analyses between circulating integrin α4⁺ ILC2 proportions and gastric function indicators or anti–Helicobacter pylori antibody levels.**

| ILC2-related parameter | Clinical variable | Correlation coefficient | Nominal  P value | FDR-adjusted q value |
| --- | --- | --- | --- | --- |
| Integrin α4^⁺^ ILC2s/ILCs | G-17 | 0.173 | 0.173 | 0.206 |
| Integrin α4^⁺^ ILC2s/ILCs | PGI | 0.160 | 0.206 | 0.206 |
| Integrin α4^⁺^ ILC2s/ILCs | PGII | 0.357 | 0.004 | 0.010 |
| Integrin α4^⁺^ ILC2s/ILCs | PGR | -0.305 | 0.014 | 0.023 |
| Integrin α4^⁺^ ILC2s/ILCs | Anti-*H. pylori* antibody | 0.497 | 0.001 | 0.005 |

Correlations were assessed using Pearson correlation analysis or Spearman rank correlation analysis, as appropriate. FDR-adjusted q values were calculated using the Benjamini–Hochberg procedure within this prespecified integrin α4⁺ ILC2 correlation-analysis family. The correlation coefficient is reported as Pearson’s r or Spearman’s ρ, according to the method used. G-17, gastrin-17; PGI, pepsinogen I; PGII, pepsinogen II; PGR, PGI/PGII ratio; FDR, false discovery rate.

**Supplementary Table S8. Exploratory comparisons of circulating ILC2-related profiles between available baseline and post-WMT samples and according to post-WMT Helicobacter pylori status.**

| Compared indicator | A group | B group | Nominal  P value | FDR-adjusted  q value |
| --- | --- | --- | --- | --- |
| ILC2s/ILCs | Baseline:  27.48 (21.16, 35.29)  n=23 | After WMT:  10.08 (6.83, 18.40) n=16 | <0.001 | <0.001 |
| Integrin α4^⁺^ ILC2s/ILCs | Baseline:  15.37 (9.12, 21.92)  n=23 | After WMT:  4.27 (2.89, 7.40)  n=16 | <0.001 | <0.001 |
| ILC2s/ILCs | Post-WMT  *H. pylori*-positive:  18.18 ± 9.50  n=7 | Post-WMT  *H. pylori*-negative:  8.10 ± 2.53  n=9 | 0.031 | 0.041 |
| Integrin α4^⁺^ ILC2s/ILCs | Post-WMT  *H. pylori*-positive:  7.18 ± 6.24  n=7 | Post-WMT  *H. pylori*-negative:  4.24 ± 1.91  n=9 | 0.269 | 0.269 |

Values are presented as mean ± standard deviation or median (interquartile range), as appropriate. Nominal P values were obtained using the independent-samples Student’s t test or Mann–Whitney U test according to data distribution. FDR-adjusted q values were calculated using the Benjamini–Hochberg procedure within this prespecified WMT-related comparison family. Baseline and post-WMT samples were not fully paired; therefore, these results were interpreted as exploratory between-group comparisons rather than longitudinal within-patient changes. WMT, washed microbiota transplantation; ILC2, group 2 innate lymphoid cell; FDR, false discovery rate.

**Supplementary Figure S1. Hypothetical model illustrating potential microbiota–ILC2 interactions in Helicobacter pylori infection and washed microbiota transplantation (WMT)-related immune responses.**

This schematic summarizes a hypothetical framework describing how microbiota alterations, microbial-derived metabolites, and epithelial signaling pathways may influence ILC2-related immune responses in the context of *H. pylori* infection and WMT exposure. Altered ILC2 profiles may be associated with circulating immune phenotypes and post-WMT *H. pylori* status. This conceptual model is based on previous evidence and the exploratory observations of the present study. The proposed pathways are not directly validated in this study, and dashed arrows indicate hypothetical relationships requiring further investigation.
